# Novel procedure of CO_2_ capture of the CaO sorbent activator on the reaction of one-part alkali-activated slag†

**DOI:** 10.1039/d1ra01353j

**Published:** 2021-03-30

**Authors:** Hao Zheng, Yan He, Yuqing Zhu, Leping Liu, Xuemin Cui

**Affiliations:** College of Chemistry and Chemical Engineering, Guangxi University Nanning 530004 P. R. China 20130017@gxu.edu.cn +86-0771-3233217; Guangxi Key Laboratory of Petrochemical Resource Processing and Process Intensification Technology, Guangxi University Nanning 530004 P. R. China; College of Chemistry and Materials, Guangxi Key Laboratory of Natural Polymer Chemistry and Physics, Nanning Normal University Nanning 530001 P. R. China

## Abstract

CaO derived naturally from limestone or dolomite is an inexpensive and widely available sorbent. Understanding the mechanisms of CaO carbonation at ambient temperature under the assistance of H_2_O is important for predicting the reaction of CaO in complex environments and designing novel CaO materials. In this study, we found that the reaction rate of alkali-activated slag is controlled by the CaCO_3_ layer on a partially carbonized CaO alkali activator. The size of the sorbent increased after the adsorption reaction and the physically adsorbed water in the pores accelerated the carbonation. The carbonation process was governed by CO_2_ diffusion. When the carbonation conversion rate reached 2–6%, the setting time increased rapidly with the increase in the carbonation rate. This is because the surface of the activator was modified by the thickened CaCO_3_ product layer, which increased the diffusional resistance and thus prolonged the setting time of the alkali-activated slag.

## Abstract 2

The proposed strategy builds a bridge between the alkali-activated cement and CaO-based CO_2_ sorbents, and this study provides a facile and cost-effective method for controlling the reaction rate of alkali-activated slag.

## Graphical abstract

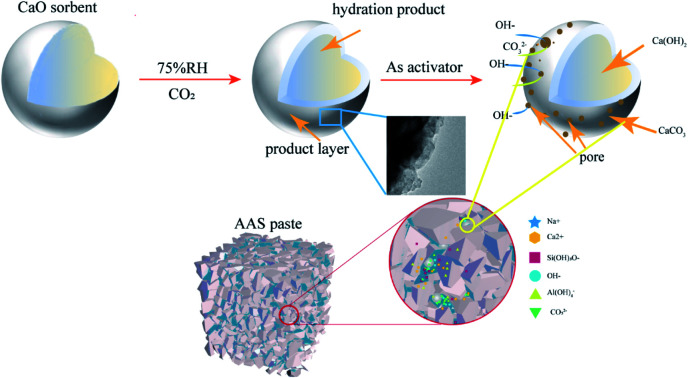

## 1. Introduction

Carbon dioxide (CO_2_) plays an important role in the Earth's ecosystem, but high CO_2_ emissions exacerbate the greenhouse effect. The traditional building materials industry produces a large amount of CO_2_, and the use of alkali-activated cement can decrease the emission of this greenhouse gas.^1^ The precursors are derived from several Si- and Al-rich minerals and industrial wastes including metakaolin, ground blast furnace slags, and fly ash.^2^ Geopolymers have been considered as environmentally friendly substitutes that can replace Portland cement in construction materials.^3^ CO_2_ capture is generally based on the use of a variety of sorbents, such as inorganic chemisorbents, MOFs, and organoamine adsorbents, which exhibited high theoretical uptake capacity, excellent catalytic performances, and generated valuable byproducts respectively.^4,5^ In particular, CO_2_ mineral sequestration deserves special attention as it is obtained directly. Du *et al.*^6^ found CO_2_ has a strong affinity with montmorillonite, indicating a potential solution for CO_2_ sequestration into a deep shale reservoir. Calcium-based sorbents (CaO, Ca(OH)_2_) are an abundant material and can be obtained easily at low cost. Theoretically, 1 g of CaO can capture 786 mg of CO_2_. In addition, this sorbent could be applied at room temperature and atmospheric pressure.^9,16^ The reaction of calcium oxide (CaO) with CO_2_ at high temperatures has great significance.^7,8^ In the presence of CO_2_, CaO transforms into a stable carbonate, which can be a useful technology for stable CO_2_ storage.^8,9^ The reaction between CaO and CO_2_ proceeds in two stages.^10–13^ In the first stage, CO_2_ reacts rapidly with CaO to form dense calcium carbonate (CaCO_3_). In the next stage, CO_2_ in the gas phase can react with CaO only by diffusing through the initially formed layer of CaCO_3_ products. The diffusion coefficients for CaO and CaCO_3_ are 0.3 and 0.03 cm^2^ s^−1^, respectively.^14^ Thus, the initial fast stage and the subsequent slow stage are controlled by the kinetics of the reaction and diffusion of CO_2_ through the CaCO_3_ product layer, respectively. CaO requires temperatures in excess of 300 °C to react with CO_2_ at a reasonable rate.^11^ At low temperatures, the carbonation of CaO requires the assistance of water vapor, and the relative humidity of the gas phase should be more than 8% to carbonize CaO to obtain CaCO_3_ and temperature affects this reaction mildly, and relative humidity affects this reaction significantly.^15^ Very recently, Moreno *et al.*^16^ used hydrated lime samples exposed for six days to atmospheres of different relative humidity (RH) of 24, 58, 75, and 100%, respectively. They found that the CO_2_-capture capacity is related to the mechanism that governs the physisorption of water on calcium hydroxide (Ca(OH)_2_) at room temperature. Although CaO derived naturally from limestone or dolomite is inexpensive and widely available, its sorption capacity declines rapidly to only 10% of its theoretical capacity after several cycles of usage owing to severe particle sintering and attrition.^17,18^ However, the costs and profits of the process would not allow it to be implemented on a large scale, unless there is a way to extend applications of adsorption products.

Quicklime is an important component in cement production; it can also be used as an activator in alkali-activated cements. Alkali-activated slag (AAS) is less energy-intensive and emits less CO_2_ than the ordinary Portland cement.^19^ Ca(OH)_2_ and CaO are more mild, price competitive, and environmentally friendly activators than sodium hydroxide and sodium silicate.^20,21^ According to a previous report, CaO has better activation potential in alkali-activated materials (AAMs) than Ca(OH)_2_, but the early strengthening of the paste was slow due to the lower pH of the system.^21^ The setting time reflects the rate of the alkali-activated reactions. To satisfy the requirements of different applications, the setting time of the paste of Portland cement was adjusted by the addition of different additives. However, the setting time of an alkali-activated slag is very short to be applied conveniently, and the conventional retarder does not work in alkali-activated cement. Therefore, efforts have been directed at controlling the setting time. Pradip *et al.*^22^ reported that OPC accelerated the geopolymerization reaction and aided the fly-ash-based geopolymer to achieve a setting time comparable to that of the conventional cement concrete. Hubler *et al.*^23^ studied the addition of calcium silicate hydrate seeds to an AAS paste, and observed an earlier and larger hydration rate peak and a much higher compressive strength after 1 d of curing. This is because the accelerating properties of the calcium silicate hydrate seeds rely on the nucleation and growth being the rate-limiting steps in the hydration process. However, there are very few studies on decreasing the hydration rate of AAMs, and no study has been reported on controlling the setting time of alkali-activated cement by modifying the activator.

CaO has been investigated extensively because of its high theoretical CO_2_ uptake capacity, as well as the extensive availability of low-cost CaO precursors.^24^ However, according to the well-known cycle performance of CaO carbonation, the sorption capacity of CaO sorbents decreases dramatically with cyclic sorption and desorption processes. It is therefore important to find a direct application of the resultant adsorption products. This study investigated the mechanism of the conversion of CaO to Ca(OH)_2_ and CaCO_3_ at ambient temperature and constant humidity (∼75% RH), and the effect of partially carbonized CaO as an activator on the reaction rate of an alkali-activated slag. This process could mitigate CO_2_ emissions *via* rapid CO_2_ capture and promote the research on AAMs.

## 2. Experimental section

### 2.1 Materials

Analytical grade CaO (4.665 m^2^ g^−1^) and Na_2_CO_3_ (XILONG Scientific Co., Ltd.) were used in this study. Granulated blast-furnace slag (GGBFS) powder from the Beihai Chengde Group Company was used as the precursor of the one-part AAMs. The GGBFS mainly consists of CaO (57.4 wt%), SiO_2_ (19.2 wt%), Al_2_O_3_ (9.5 wt%), and MgO (3.3 wt%). This slag has a Blaine fineness of 3973 cm^2^ g^−1^ and an average particle size, *d*_50_ of 11.3 μm, as determined by laser diffraction.

### 2.2 Experimental procedure

#### 2.2.1 Hydration–carbonation

In this study, controlled humidity environments were achieved using supersaturated aqueous NaCl solutions, which yielded an RH of 75% in a closed vessel (56 L). The CaO sorbent (56 g, 1 mol) was spread at the bottom of a plastic dish. The air was pumped out and replaced with CO_2_ (2.24 L) (purity, >99%) and nitrogen (purity, >99.999%) at an absolute pressure of 1 bar. After different reaction times, the sorbent was extracted from the vessel and immediately used for characterization and activation.

#### 2.2.2 Preparation of one-part alkali-activated slag paste

In order to maintain the alkaline content consistently, alkali-activated slag pastes were produced with an activator dose of 6.8 g of Na_2_CO_3_ + sorbent (3.6 g of CaO + *n*H_2_O + *m*CaCO_3_) per 100 g of slag, and a water/(slag + activator) mass ratio of 0.40. The detailed compositions are provided in Table S1 in the ESI.† The GGBFS powder was carefully dry-mixed with the CaO and Na_2_CO_3_ powders for 10 min and then mixed with water. The mixture was dispersed using a disperser at 800 rpm for 3 min before it was injected into the mold.

### 2.3 Characterization

To investigate the change in the crystalline phase, X-ray diffraction (XRD) was conducted on a Rigaku Miniflex 600 diffractometer equipped with a Ni filter and Cu-Kα radiation source. The equipment was operated at 40 kV and 15 mA, and XRD patterns were collected in the 2*θ* range of 5–80° with a step of 0.02° at the rate of 5°/min. Scanning electron microscopy (SEM) was performed on a field-emission scanning electron microscope (SU 8220; Japan Hitachi Limited Company) at an accelerating voltage of 10 kV. Transmission electron microscopy (TEM) was performed on an FEI TECNAI G2 F30 field-emission scanning/transmission electron microscope equipped with an energy-dispersive X-ray spectrometer and a Gatan Tridiem imaging filter at 300 kV. The samples used for TEM were ground and dispersed in acetone.^10^

The hydration, carbonation, and total conversion of the treated samples were determined by the calcination of the reacted material using a thermogravimetric analyzer (TGA; METTLER TOLEDO TGA 2).^25^ In each test, 20–30 mg of the material was heated from ambient temperature to 1000 °C at a heating rate of 10 °C min^−1^ under a nitrogen flow (rate: 50 mL min^−1^). The accuracy of the TGA is ±0.001 mg.

The initial and final setting times of the alkali-activated slag paste were measured according to ASTM C191 (Vicat needle penetration test).^26^ The Vicat measurement of the initial setting time was started when the dried materials with the activating solution were mixed and then, the measurements were conducted after different intervals until the Vicat needle penetration was equal to or less than 25 mm (1 in). The final setting time was measured from the time that the activating solution and dry material were contacted with the Vicat needle until the needle could not penetrate the surface of the paste sample. The compressive strength of the sample was measured using an electronic universal testing machine (DNS100); at the age of 28 days, the tests were conducted on 40 mm cubic specimens. These cubic specimens were tested at a load rate of 0.5 MPa s^−1^ in accordance with ASTM C109.

## 3. Results and discussion

### 3.1 Mechanisms of CaO hydration and carbonation

**Fig. 1:**
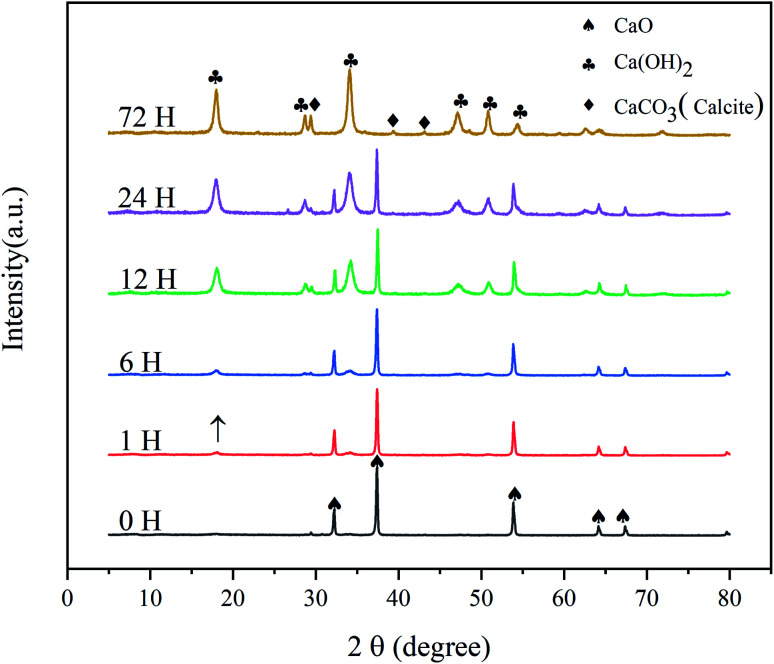
X-ray diffraction patterns of CaO activators at different times.

The XRD patterns of the CaO sorbents reacted with CO_2_ and H_2_O for different times are shown in Fig. 1. All the samples were reacted in a sealed container at room temperature (20 °C, 75 ± 6% RH), and the initial amount of CO_2_ was maintained at 10% of that of CaO. The patterns obtained at later times revealed the gradual disappearance of CaO (PDF# 37-1497). After a reaction time of 1 h, the diffraction peak of calcium hydroxide (Ca(OH)_2_; 2*θ* = 18.065°) appeared, but its intensity was very weak. This result indicates that the hydration of CaO had just begun; the surface of CaO absorbed water molecules in the gas phase to form Ca(OH)_2_, and the conversion rate was low. After 6 h, the peaks of Ca(OH)_2_ (PDF# 76-0571) were further enhanced. At the same time, the diffraction peaks of calcite (CaCO_3_; 2*θ* = 29.409°) appeared with a low intensity. This result indicates the chemical adsorption of CO_2_ on the surface of CaO to generate CaCO_3_. After exposure for 12 h, the diffraction peaks of Ca(OH)_2_ intensified, indicating a significant increase in the content of Ca(OH)_2_. After 24 and 72 h, the XRD patterns of the commercial CaO sample hydrated and carbonized at room temperature exhibited much stronger diffraction peaks at similar positions, and the peaks of the calcite phase (PDF# 83-0578) became more obvious during 24–72 h.

**Fig. 2:**
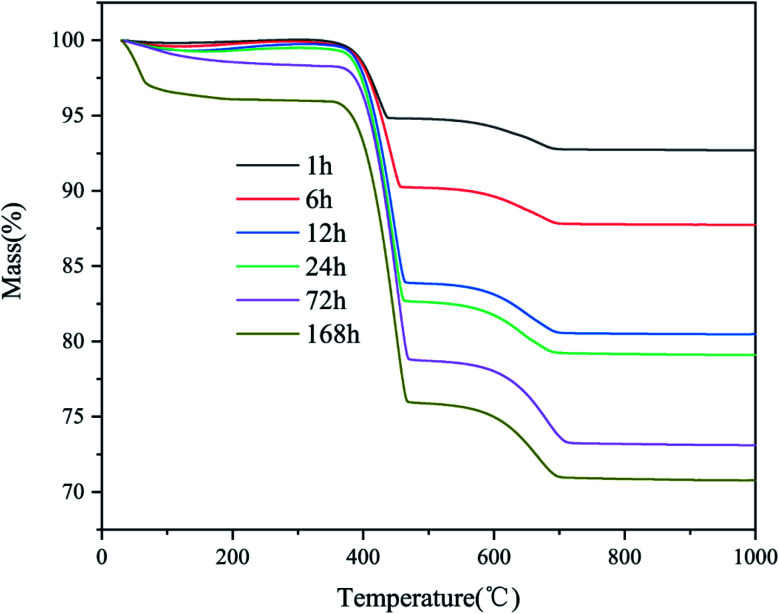
Thermogravimetric analysis of CaO samples exposed to CO_2_ in a sealed container for different times.

The TGA curve in Fig. 2 reveals the weight loss behavior of the products. The weight loss of the products can be divided into two main regions: 350–500 °C and 600–800 °C. In the first region, the weight loss was due to the decomposition of the chemically bound water of calcium hydroxide. The weight loss in the second region is due to the decomposition of calcite. The water molecules in the gas phase constantly contacted the surface of the CaO particles and reacted rapidly to form calcium hydroxide. At the same time, CO_2_ reacted with the particles to form CaCO_3_ with the assistance of H_2_O.

**Fig. 3:**
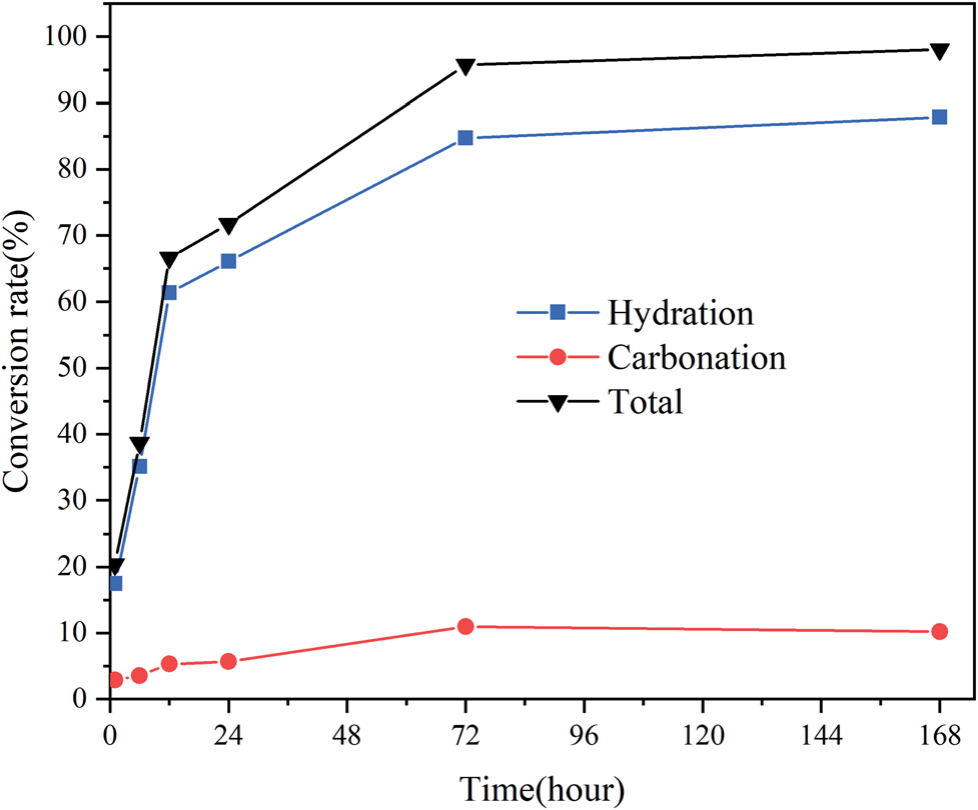
Carbonation and hydration conversion rates of CaO exposed to CO_2_ and 75% RH in a sealed container for different times.

Fig. 3 presents the hydration, carbonation, and total conversion of CaO over time. As the amount of CO_2_ was fixed at ∼10% of the amount of CaO, there was a balance when the carbonation rate increased to ∼10%. An obvious weight loss peak was observed before 100 °C in the TGA curve for the samples stored for 168 h, owing to the loss of free water adsorbed to the micropores of the products after complete hydration and partial carbonization. According to the Kelvin equation, if the surface curvature is concave, the actual vapor pressure of the liquid phase will be higher than the saturated vapor pressure at a flat surface, owing to the surface tension of the solid–liquid interface. Thus, because CaO is hydrophilic, the smaller the pore size is, the easier the condensation of moisture from the air in the pores is. The pore size of commercial CaO is less than 10 nm. The applicability limit of the Kelvin equation is also believed to be until the confinement of a few molecular layers.^27^ According to the Kelvin–Laplace equation, at 75% RH, water can condense in all the pores with radii smaller than ∼5 nm. Owing to the condensation of water vapor in the micropores and mesopores of CaO, the CaO hydration reaction transforms into a condensation-controlled microscopic liquid–solid reaction. This explains the subsequent slow hydration rate of CaO.

**Fig. 4:**
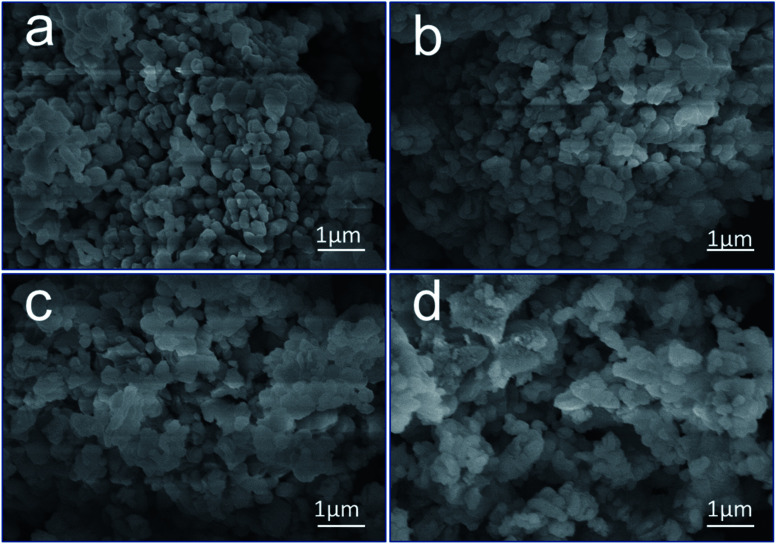
SEM images of CaO after exposure to moisture and CO_2_ for (a) 0 h, (b) 6 h, (c) 12 h, and (d) 72 h.

Fig. 4 displays the microstructure of CaO at different times. Fig. 4(a) reveals that CaO has a compact structure with a smooth surface. After hydration and carbonization, the surface became rough, and the surface area increased, as suggested by the morphologies in Fig. 4(b)–(d), indicating that the pores of CaO were filled. This result is consistent with the results of the conversion (Fig. 3); the sample was almost completely hydrated and carbonated by 10% after 72 h of exposure. CaO was hydrated to form Ca(OH)_2_, and the CaCO_3_ layer was deposited on the outer surface of CaO.

**Fig. 5:**
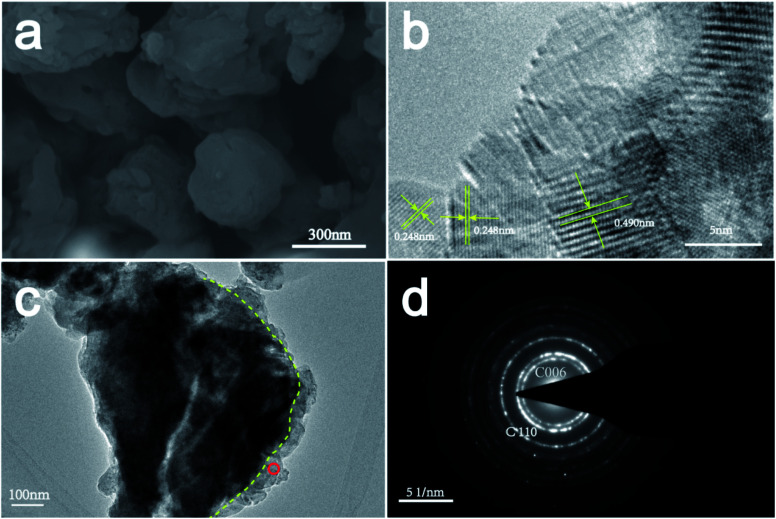
(a) SEM and (b and c) TEM images of the activator after 72 h of treatment and (d) selected-area electron diffraction pattern of the product layer.

The SEM and TEM images of the CaCO_3_ layer are shown in Fig. 5(a)–(c). The (006) and (110) crystal planes of CaCO_3_ were observed in the electron diffraction pattern (Fig. 5(d)), in agreement with the XRD results (Fig. 1). Additionally, the interplanar distance of the outer layer of the sorbent was measured to be 0.248 nm, corresponding to the (110) plane of calcite (CaCO_3_). In contrast, the interplanar distance of the core of the sorbent was measured to be 0.490 nm, which corresponds to the (001) plane of Ca(OH)_2_. These results indicate that the CaCO_3_ layer adhered on the external surface of the hydration product (Ca(OH)_2_).

Next, we investigated how the hydration and carbonation reactions occurred simultaneously and how the RH affected the carbonation rate of CaO at low temperatures. Beruto and Moreno speculated that a thick multilayered adsorbed film of H_2_O was present at the pores of Ca(OH)_2_, which acted like a bidimensional liquid-like interface that facilitated the reaction of CO_2_ with Ca(OH)_2_. According to them, the carbonation mechanism of Ca(OH)_2_ at low temperatures was mediated by the physisorbed water.^28,29^ The following reaction mechanism was proposed:

First, CaO undergoes partial hydration.

1 CaO + H_2_O ⇄ Ca(OH)_2_

Then, Ca(OH)_2_ partially dissolves in the adsorbed water layers and dissociates, and the moisture in the air condenses in the pores, as described by the Kelvin equation.

2 Ca(OH)_2_(s) ⇄ Ca^2+^(aq) + 2OH^−^(aq)

Next, carbon dioxide dissolves in the physically adsorbed water layer.

3 CO_2_(aq) + H_2_O(aq) ⇄ H_2_CO_3_(aq)

Partial ionization of carbonic acid occurs.

4 H_2_CO_3_(aq) ⇄ H^+^(aq) + HCO_3_^−^(aq)

5 HCO_3_^−^(aq) ⇄ H^+^(aq) + CO_3_^2−^(aq)

However, if the alkalinity of water is sufficiently elevated, CO_2_(aq) can also react with hydroxyl ions to directly generate bicarbonate ions.

6 CO_2_(aq) + 2OH^−^ (aq) ⇄ CO_3_^2−^(aq) + H_2_O

Further, Ca(OH)_2_ has a larger molar volume than CaO, and the porosity of the grains decreases due to the formation of CaCO_3_. The released water migrates into the internal pores because the affinity of water of Ca(OH)_2_ is bigger than that between water and CaCO_3_. It reacts preferentially with CaO or enters the internal pores of Ca(OH)_2_.

7 Ca^2+^(aq) + CO_3_^2−^(aq) ⇄ CaCO_3_(s)

Finally, after the concentration of CO_3_^2−^ and Ca^2+^ reaches the solubility product of CaCO_3_, CaCO_3_ precipitates and the carbonation layer deepens. The carbonation rate did not decrease obviously with the decrease in the CO_2_ concentration, indicating that the reaction was controlled by CO_2_ diffusion through the product layer. The reaction of Ca(OH)_2_ is a zero order reaction with respect to gas-phase CO_2_ concentration.^12^

After the hydration reaction of CaO is completed, free water begins to accumulate in the pores until a new equilibrium is established. In the absence of CO_2_, the conversion of Ca(OH)_2_ to CaCO_3_ ceases.

### 3.2 Mechanism of carbonation on paste properties

**Fig. 6:**
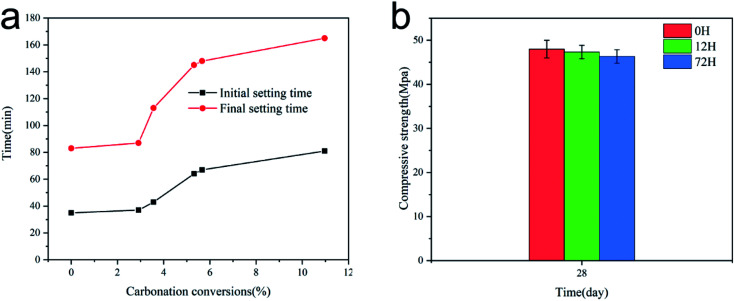
Setting time and compressive strength of AAS pastes.

Fig. 6(a) depicts the effect of carbonation rate on the setting time of AAS pastes. The setting time of the paste was measured at the temperature of 20 ± 2 °C. It has been shown that the higher the carbonation conversion rate in the paste is, the slower the rate of setting is. Alkali-activated slag pastes containing untreated CaO as the activator exhibit rapid rate of chemical reactions. The initial setting time and final setting time of the paste with untreated CaO were 35 min and 83 min, respectively. As shown in Fig. 6(a), when the carbonation rate reached 2–6%, the setting time increased rapidly with the increase in the carbonation rate. Thus, the precipitation conversion reaction was controlled by the formation of CaCO_3_ at the pores and surface of CaO/Ca(OH)_2_. During this reaction stage, CaCO_3_ grew on the surface of the CaO grains in a dispersed manner and then expanded to form a continuous layer on the external surface of the unreacted sorbent. Thereafter, the so-formed CaCO_3_ wrapped the core, and the contact sites of the reaction were reduced significantly.

Additionally, when the partially carbonized activator was mixed with water, calcium hydroxide, which is slightly soluble in water, released Ca^2+^ into the solution. The released Ca^2+^ reacted with CO_3_^2−^ in the solution and promoted the release of OH^−^ at the same time. It can be speculated that the reaction of Ca(OH)_2_ in the carbonate solution involves several steps.

8 CaO(s) + H_2_O(aq) → Ca(OH)_2_(s)

The remaining CaO was hydrated in the solution.

9 Ca(OH)_2_(s) ⇄ Ca^2+^(aq) + 2OH^−^(aq)

Ca(OH)_2_ partially dissolved and dissociated in the solution.

10 Na_2_CO_3_(aq) → 2Na^2+^(aq) + CO_3_^2−^(aq)

Na_2_CO_3_ dissolved and ionized in the solution.

11 Ca^2+^(aq) + CO_3_^2−^(aq) ⇄ CaCO_3_(s)

Ca^2+^ and CO_3_^2−^ diffused in the liquid–solid layer. Upon coming in contact, Ca^2+^ and CO_3_^2−^ generated CaCO_3_, which crystallized and precipitated in the solution. Reaction (4) promoted the shift in the equilibrium of reaction (2) to the right.

Therefore, the present results indicate that the carbonation rate of Ca(OH)_2_ in the solution was affected by the CaCO_3_ product layer. When the pH of the solution increased, OH^−^ induced the fracture of –Ca–O– and –Si–O– on the slag surface and released Ca^2+^ and SiO_4_^4−^, which nucleated in the solution to form a large number of hydration products. However, the formation rate of OH^−^ was reduced by the formation of a CaCO_3_ layer on the surface of Ca(OH)_2_. Consequently, the dissociation rate of the slag and the formation rate of the hydration products decreased. The initial and final setting times increased by 131 and 98%, respectively, when the carbonization rate of the activator reached ∼10%.

Fig. 6(b) shows the compressive strength of the specimens added with sorbents treated for different times after curing for 28 days. The compressive strengths of the test specimens with sorbents treated for 0, 12, and 17 h were 48.0, 47.3, and 46.3 MPa, respectively, as determined using a universal testing machine. These values meet the technical standard of GB 175-1999 42.5 of the industry. The compressive strength results suggested that the activator did not have a significant effect on the compressive strength of the cured specimens, although the setting time was affected. It can be concluded that CaCO_3_ does not affect the geopolymerization process, apart from delaying it.

**Fig. 7:**
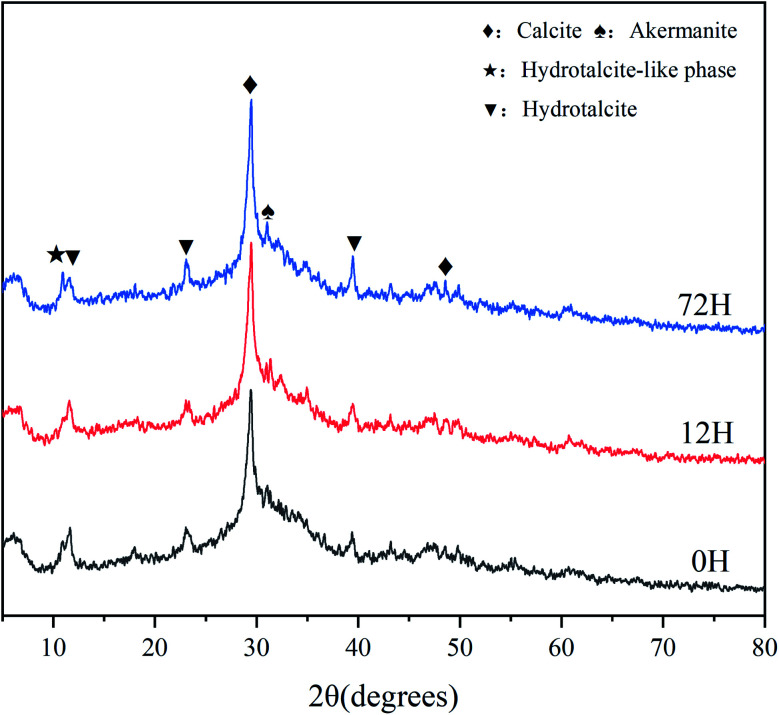
XRD patterns of the paste of AAS at 28 d.

In the XRD patterns (Fig. 7), the broad and diffuse peak of C–A–S–H (2*θ* = 25–35°) is evident in all hardened sample, this is one of main hydration products of AAS.^21^ The diffraction peaks of hydrotalcite-like phase (2*θ* = 10.94°) and hydrotalcite (2*θ* = 11.27°) are also observed for all paste, which has also previously been found in other AAS studies. Strong peaks of calcite (2*θ* = 29.4°) are seen for different samples, which associated with the reaction (8)–(11) of solid activator. In addition, the calcite can also generate from carbonation of C–A–S–H. Moreover, akermanite is exist in all sample, which has been reported in different AAS systems that with high levels of magnesium.^30^

## 4. Conclusions

Carbon negative emission technologies are strong active measures for mitigating climate change. It is important to control the setting time of the AAS at the ambient temperature for understanding the mechanisms of alkali-activated reactions. This study investigated how CO_2_ and H_2_O were captured by CaO in a batch reactor in a controlled humidity environment. The hydration and carbonation of the sorbent were catalyzed by the physisorption of water in the pores of Ca(OH)_2_ at room temperature. A significant reduction in the CO_2_ concentration in the reactor did not have a noticeable effect on the carbonation rate. This is because the carbonation process is governed by the diffusion of CO_2_. After the formation of the CaCO_3_ product layer on the outer surface of the CaO grains, the diffusional resistance to CO_2_ increased. This decreased the rate of geopolymerization, and the initial and final setting times of alkali-activated slag increased by 131 and 98%, respectively.

## Conflicts of interest

The authors declare that they have no known competing financial interests or personal relationships that could have appeared to influence the work reported in this paper.

## Supplementary Material

RA-011-D1RA01353J-s001
