# Supplementary material for: Novel procedure of CO2 capture of the CaO sorbent activator on the reaction of one-part alkali-activated slag
Source: RSC Adv. 2021 Mar 30;11(21):12476–83. doi: 10.1039/d1ra01353j (PMC8696962; doi:10.1039/d1ra01353j)
Supplement: RA-011-D1RA01353J-s001 [file RA-011-D1RA01353J-s001.pdf]

Electronic Supplementary Material (ESI) for RSC Advances.
This journal is © The Royal Society of Chemistry 2021

Table. 1. Mix proportions for AAS paste

	Slag	H_2O	Na_2CO_3	sorbent
CN 0 H	100	44	6.8	3.60
CN 1 H	100	44	6.8	4.00
CN 6 H	100	44	6.8	4.26
CN 12 H	100	44	6.8	4.71
CN 24 H	100	44	6.8	4.80
CN 72 H	100	44	6.8	5.49

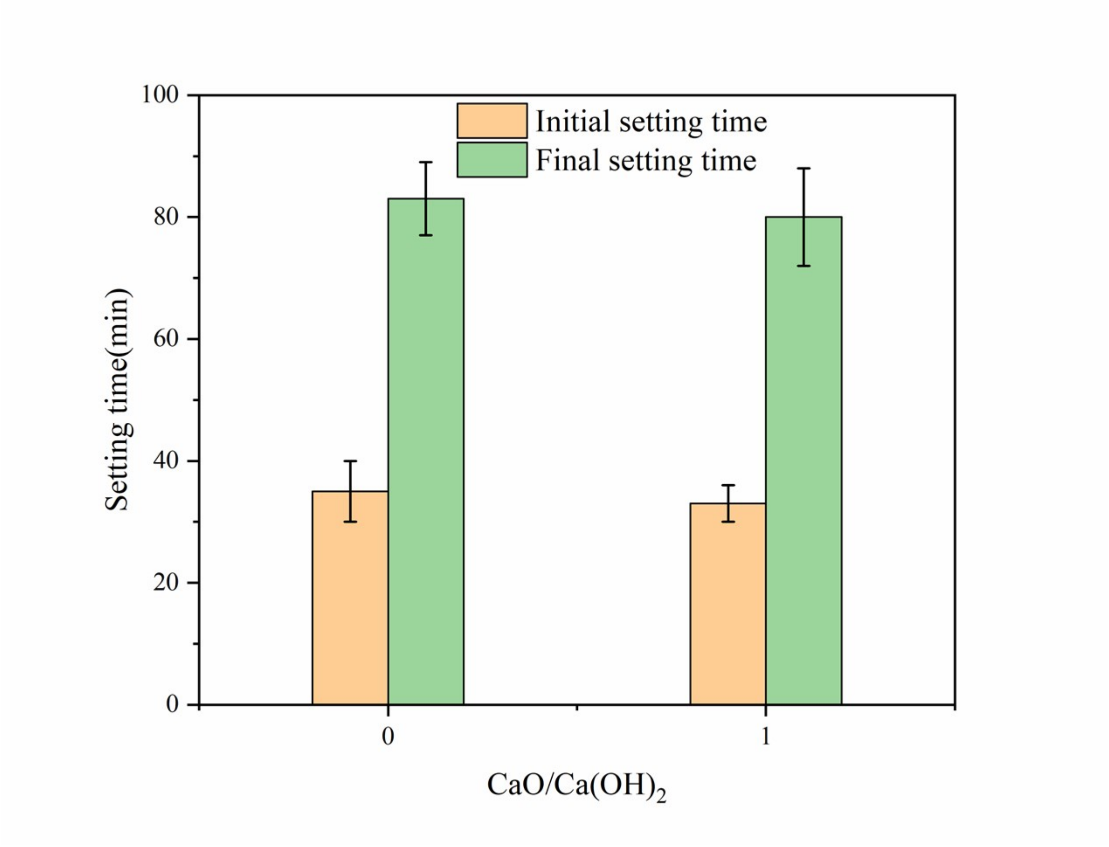

Figure.s.1 The setting time of the AAS paste activated by the pure $CaO/Ca(OH)_2$.

Figure.s.1 shows that the conversion of calcium oxide to calcium hydroxide has negligible effect on the setting time of the AAS paste.

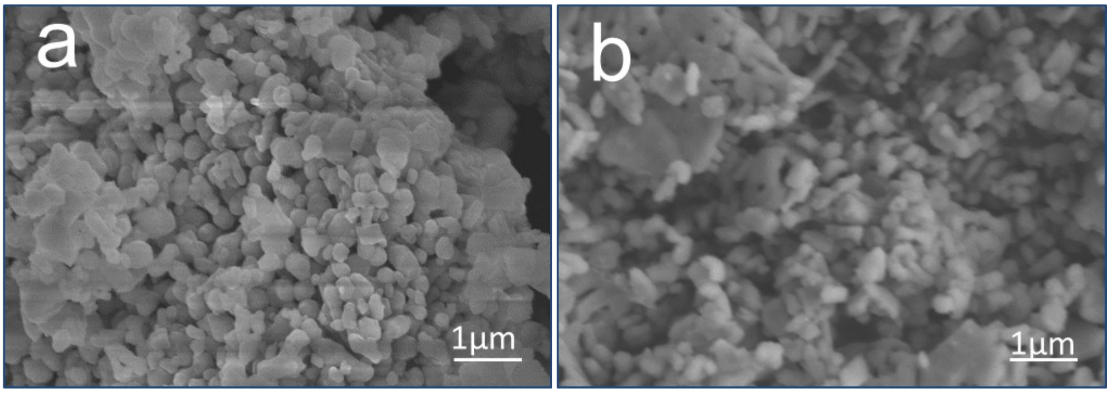

Figure.s.2 SEM image of different kinds of CaO for a (analytical grade CaO), b (calcined limestone)

Figure.s.2 shows different kinds of calcined CaCO3 particle possesses a similar structure.
